# Three-dimensional reconstruction on cell level: case study elucidates the ultrastructure of the spinning apparatus of *Embia* sp. (Insecta: Embioptera)

**DOI:** 10.1098/rsos.160563

**Published:** 2016-10-12

**Authors:** Sebastian Büsse, Thomas Hörnschemeyer, Christian Fischer

**Affiliations:** 1 Department of Functional Morphology and Biomechanics, Institute of Zoology, Christian-Albrechts-Universität zu Kiel, Am Botanischen Garten 1–9, 24118 Kiel, Germany; 2 Senckenberg Gesellschaft für Naturforschung, Senckenberganlage 25, 60325 Frankfurt, Germany; 3 Department of Morphology, Systematics and Evolutionary Biology, J.- F.- Blumenbach Institute for Zoology and Anthropology, Georg-August-Universität Göttingen, Berliner Strasse 28, 37073 Göttingen, Germany

**Keywords:** serial block-face scanning electron microscopy, ultrastructural research, dermal glands, spinning apparatus, step-by-step protocol

## Abstract

Spinning is a phenomenon not only present in spiders, but also in many other arthropods. The functional morphology and complexity of spinning organs is often poorly understood. Their elements are minute and studying them poses substantial methodological difficulties. This study presents a three-dimensional reconstruction of a silk gland of *Embia* sp. on cellular level, based on serial sections acquired with serial block-face scanning electron microscopy (SBFSEM) to showcase the power of this method. Previous studies achieved either high resolution to elucidate the ultrastructure or satisfying three-dimensional representations. The high-resolution achieved by SBFSEM can be easily used to reconstruct the three-dimensional ultrastructural organization of cellular structures. The herein investigated spinning apparatus of Embioptera can be taken as an example demonstrating the potential of this method. It was possible to reconstruct a multinucleated silk gland containing 63 nuclei. We focused on the applicability of this method in the field of morphological research and provide a step-by-step guide to the methodology. This will help in applying the method to other arthropod taxa and will help significantly in adapting the method to other animals, animal parts and tissues.

## 1. Background

The biological context in which arthropods use silk is very broad, ranging from prey capture through mating to the construction of domiciles [1,2]. At the same time, spinning apparatuses are diverse with respect to their morphology and can be present on a wide range of body parts [1,2]. Spiders, for example, use spigots that are located at their opisthosoma to produce a distinct thread [3], whereas the spinning organs of webspinners (Embioptera) are located in their forelegs [4,5]. Based on phylogenetic cladograms spinning apparatuses evolved many times independently within the arthropods [2].

In insects, silk glands can be derived from Malpighian tubules, labial glands or dermal glands [6], their origin, occurrence and use is as diverse as insects themselves [2,7–10].

The most distinctive character of webspinners (Embioptera) is the silk-spinning apparatus present in the enlarged basitarsomeres of the forelegs. Each multinucleated silk gland (class III after [2]) is enclosing a reservoir, which is capable of storing the produced silk. A secretory duct opens into hollow, cuticular hair-like tubes (silk-ejectors) on the ventral surface of the basitarsomere [4,5,11–18].

The analysis of functional morphology heavily relies on investigating the behavioural context, morphology and the function of a structural complex. The embiopteran spinning apparatus has already been studied to some extent [4,5,11–18]. Morphological studies, however, ask questions with respect to texture, composition and organization of a structural character. Morphological techniques such as light microscopy, transmission electron microscopy (TEM), scanning electron microscopy (SEM) and more recently microcomputed X-ray tomography (µCT) have their distinct strengths and limitations. Often three-dimensional representations provide a significantly better understanding of complex morphological structures. In the past, manual dissection, SEM and µCT have been the techniques of choice with regard to three-dimensional representation and user friendliness. There are also numerous fine examples of three-dimensional representations of morphological characters based on histological and TEM studies. However, both techniques are very time consuming. With respect to ultrastructural studies that require a resolution provided by electron microscopy, serial block-face scanning electron microscopy (SBFSEM) is a promising recent addition to the morphologist's toolbox [19–25].

SBFSEM combines high resolution close to what the TEM provides with user friendliness. An embedded sample is mounted into an SEM equipped with an automated ultramicrotome. This combination of units produces perfectly stacked high-resolution images [21,22]. These images can be used to reconstruct a three-dimensional representation of the sample [26] depicting all structures on a cell level.

We here present an ultrastructural study, elucidating the morphology of the multinucleated spinning apparatus of *Embia* sp. We provide a three-dimensional reconstruction of cell organelles such as the nucleus, mitochondria and the Golgi apparatus of the spinning apparatus. Furthermore, we discuss the potentials of this method and provide a step-by-step protocol from an insect morphologist's point of view.

## 2. Method

### 2.1. Material

Six females of *Embia* sp. Latreille, 1829 (Embiidae) were collected in Ibiza, Spain. All regulations concerning the protection of free-living species were followed (see also Ethics section). Four of these specimens were sectioned and compared. One of these specimens was used for producing the three-dimensional reconstructions presented herein (see Three-dimensional reconstruction paragraph).

### 2.2. Electron microscopy

Specimens were studied using an FEI Quanta 250 FEG combined with a GATAN^®^ 3View system, resulting in an SBFSEM. In total, 1397 sections of 80 nm thickness were cut, and microphotographs with 4076 pixel × 4076 pixel each were taken, from the specimen presented in this study.

Owing to the distinctive requirements of the SBFSEM and the characteristics of insect tissues, a particular protocol was developed (see also electronic supplementary material, S1). In general, investigations of tissues using SBFSEM require a much stronger staining with heavy metals than protocols for TEM. A higher contrast of membranes to the surrounding areas is essential, as SBFSEM has to be operated with a low accelerating voltage of 2.5 kV to avoid charging.

In order to achieve an optimal penetration of chemicals into the tissue each procedure of the protocol was performed using a rotary disc or a rotary plate. Specimens were prefixed with 2.5% glutaraldehyde for 90 min at 4°C. Rinsing in cacodylate buffer (4°C) five times preceded the postfixation with a double treatment of OsO_4_. The first OsO_4_ treatment was performed for 60 min at 4°C (eight drops of 2% OsO_4_ in ddH_2_O, 16 drops of rinsing buffer, adding a spatula tip of potassium hexacyanoferrate). After rinsing with ddH_2_O (five times), a treatment of the tissue with freshly prepared TCH-solution followed. An amount of 0.1 g thiocarbohydrazide (TCH) was dissolved in 10 ml ddH_2_O, carefully stirred, put in an oven for 1 h at 60°C, and finally filtered through a filter with 0.22 µm pores. Specimens were treated in TCH-solution for 20 min at 20°C and subsequently rinsed in ddH_2_O five times at 20°C. The second OsO_4_ treatment (eight drops of 2% OsO_4_ in ddH_2_O, 16 drops of rinsing buffer) was performed for 30 min at 20°C. To improve the contrast the second OsO_4_ treatment can be extended to an overnight treatment without harming the tissue fine structure. Therefore, we advise to adapt the second OsO_4_ treatment to sample condition and preferred staining intensity.

Before staining en bloc, specimens were again rinsed five times in ddH_2_O (20°C) and transferred to new jars. Overnight specimens were treated in an aqueous 2.5% uranyl acetate solution at 4°C. After rinsing with ddH_2_O (five times) at 20°C, a treatment with lead citrate for 30 min at 60°C followed. After rinsing in ddH_2_O (five times), specimens were dehydrated in a graded ethanol series (20%, 50%, 70%, 90%, ‘100%’), remaining in each step for 20 min. Finally, specimens were transferred for 10 min into 100% acetone at 20°C.

To meet the requirements of maximum stability owing to the permanent impact of electrons on the specimen, Durcupan^®^ was used as embedding medium. Specimens were subsequently treated in graded Durcupan solutions (25% + 75% acetone, 50% + 50% acetone, 75% + 25% acetone), remaining 60 min in each step at 20°C. Finally, specimens were transferred in 100% Durcupan and remained overnight at 20°C. The next day, specimens were transferred to fresh 100% Durcupan and mounted on specimen pins. A transparent plastic tube with the same diameter as the pin was imposed over the pin. A syringe funnelled the embedding medium into the plastic tube. Finally, the specimens were transferred and aligned. Durcupan was allowed to polymerize in an oven at 60°C for at least 48 h. After polymerization, the plastic tube was cut off using a razor blade. Eventually, the specimen bloc was trimmed and sputtered with gold using a Balzers SPC030 sputter coater before mounting in the SBFSEM.

### 2.3. Three-dimensional reconstruction employing Amira^®^

Before using the obtained microphotographs for reconstruction in Amira^®^, they were processed in Adobe Photoshop^®^ CS3 (Adobe System Inc.) applying conversion to greyscale, Gaussian blur with a radius of 0.9 pixel and level adjustment.

Three-dimensional segmentation, processing and visualization of the data were done with Amira^®^ 5.4.3. (FEI SAS, France, www.vsg3d.com).

After evaluating data stacks of four specimens with between 800 and 1500 images of the complete spinning tarsus, we chose a specimen represented by 1397 successive images owing to tissue characteristics and preservation quality (raw data at dryad: doi:10.5061/dryad.vk3fg). The time necessary for the entire segmentation process highly depends on the experience and skills of the researcher. More detailed information on reconstruction, segmentation and visualization can be found for example in Büsse *et al*. [27].

## 3. Results

**Figure 1.**
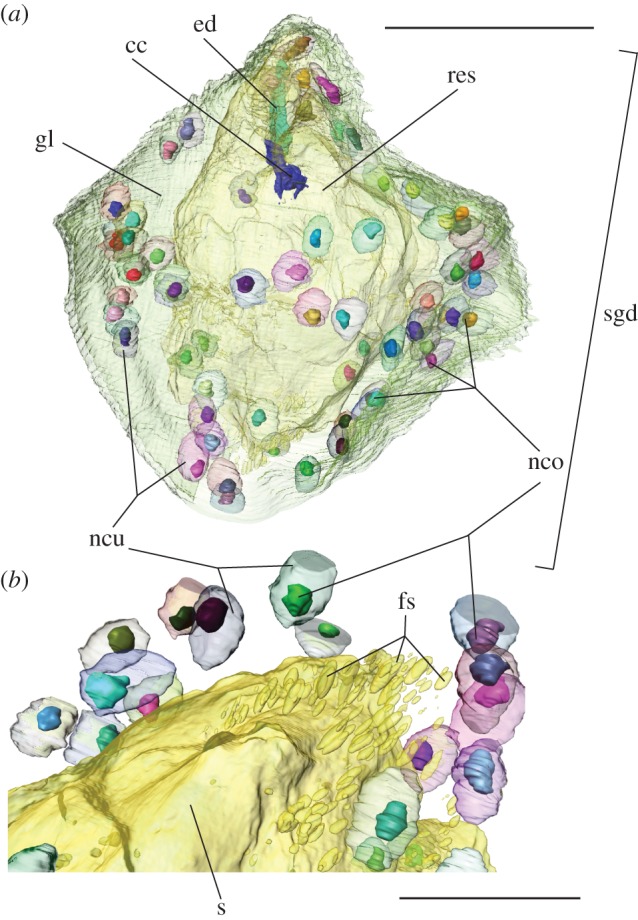
(*a*) Three-dimensional representation of a single spinning gland in the foretarsus of a female *Embia* sp. Depicted are the syncytial gland cell, its nuclei and nucleoli, reservoir, canal cage and ejection duct. (*b*) Detail shows freshly produced spinning secretion. cc, canal cage; ed, ejection duct; fs, freshly produced secretion; gl, syncytial gland cell; nco, nucleolus; ncu, nucleus; res, gland reservoir; sgd, spinning gland; s, secretion.

The overall morphology of a spinning gland in Embioptera consists of three major structural units: the reservoir, a syncytial gland cell and the ejection apparatus [5]. The reservoir is a cuticular structure in the centre of the gland (figure 1). The syncytial gland tissue surrounds each reservoir (figure 1*a*). These syncytial cells produce liquid silk, which is released into the reservoir for storage (figure 1*b*). The syncytium that we studied in most detail contained 63 nuclei.

**Figure 2.**
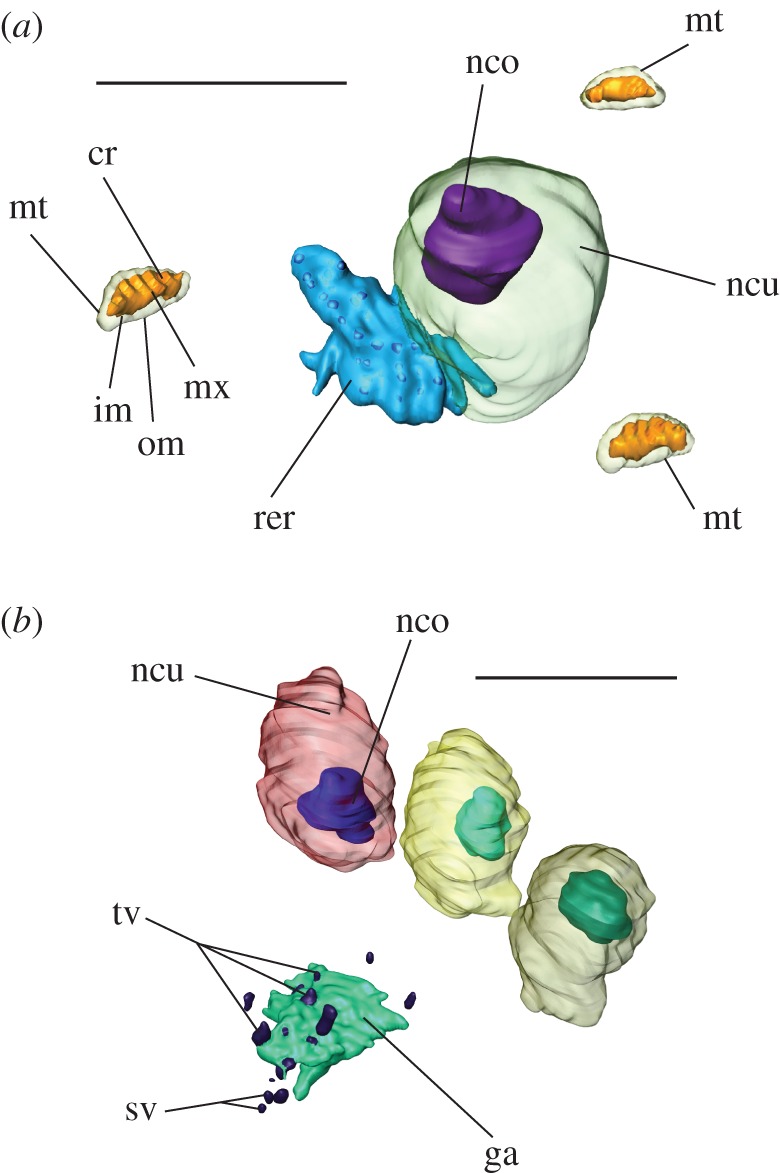
Nuclei and cell organelles within the syncytial spinning gland cell of *Embia* sp. (*a*) Single nucleus of a syncytial gland cell with associated endoplasmic reticulum and mitochondria. (*b*) Spatial arrangement of four nuclei and a Golgi apparatus. cr, cristae of mitochondrium; ga, Golgi apparatus; im, inner mitochondrial membrane; mt, mitochondrium; mx, mitochondrial matrix; nco, nucleolus; ncu, nucleus; om, outer mitochondrial membrane; rer, rough endoplasmic reticulum; sv, secretory vesicle; tv, transport vesicle.

The mitochondria can be identified by their typically shaped cristae, their inner and outer membrane, as well as their matrix (figure 2*a*). We decided to leave the mitochondria uncounted, because the number of mitochondria in a single glandular syncytium would be skyrocketing. In general, counting is feasible based on the results obtained from SBFSEM. The endoplasmic reticulum can be identified owing to its sack or tube-like cisternae. The endoplasmic membranes merge with the outer nuclear membrane (figure 2*a*). Transport vesicles produced in the endoplasmic reticulum can be seen to fuse with the Golgi apparatus on its *cis* face (figure 2*b*). Modified secretory vesicles exit the Golgi apparatus on the *trans* face (figure 2*b*).

**Figure 3.**
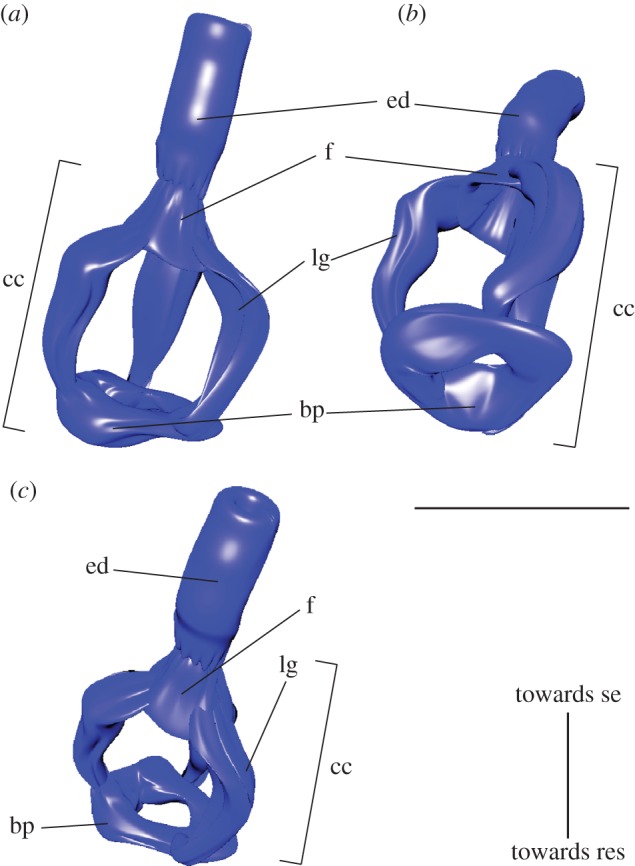
The canal cage of the ejection apparatus is a unique feature of Embioptera. It resembles an egg whisk and is supposed to collect the spinning secretion. Panels (*a–c*) provide views from different angles. bp, base plate; cc, canal cage; ed, ejection duct; f, funnel; lg, ledge; res, gland reservoir; se, secret ejection.

The three-dimensional reconstruction of the ejection apparatus reveals its unique structure. Three sections of the ejection apparatus can be identified: the silk ejector, the ejection duct and the canal cage, a term introduced to embiopteran morphology by Barth [14]. The canal cage is protruding into the reservoir and is composed of a basal plate, three ledges and a funnel—resembling an egg whisk (figure 3). The basal plate has a nearly circular opening in its centre. Three ledges connect the basal plate with the funnel, which finally opens into the ejection duct. The basal plate is oriented towards the reservoir. The canal cage is most likely homologous to the end apparatus of dermal insect glands class III [5]. The ejection duct opens at the ventral side of the basitarsomere with a hair-like silk ejector (cf. fig. 4*b,c* in Büsse *et al*. [5]) where the silk is released for spinning (raw data at dryad: doi:10.5061/dryad.vk3fg).

## 4. Discussion

Originally, SBFSEM was developed more than 10 years ago and employed to investigate the ultrastructure of nervous system tissues [19]. Today, the very time-consuming labour of sectioning and imaging of such sections as in TEM investigations can be consigned to the automated process of the SBFSEM. In the past, most of the ultrastructural research focused on model organisms, and those characters have been little used in cladistic analyses. However, some promising studies clearly demonstrate that ultrastructural character complexes can be used with great success for resolving phylogenetic relationships among taxa, e.g. ultrastructure of the rhabdom of Heteroptera [28] or the sperm structure of Embioptera [29].

A comparison of morphological techniques reveals the potentials as well as the limits of SBFSEM. For more detailed information on other morphological methods, we refer to Friedrich *et al*. [21].

### 4.1. Light microscopy

*Dissection* is one of the traditional morphological methods and can enable a fast and reliable overview of insect anatomy. One main advantage is the empirical simulation of a function by moving a structure, e.g. pulling a muscle with forceps. However, small insects are difficult to investigate. The viable size is limited to approximately 2 mm [21]. Furthermore, dissection is invasive, and small structures can be overlooked or even destroyed. Employing µCT-techniques, Büsse *et al.* [30], for example, were able to depict nine formerly unnoted muscles in dragonfly larvae (Insecta: Odonata). Previous studies relied on dissections only, missing these muscles owing to their small size and unexpected attachment points.

*Histology* is an often-used tool to study insect morphology, as the specimens are often too small to carry out a dissection providing sufficient details. Following a complex fixation protocol, the specimens are embedded and subsequently cut into semi-thin or thick sections [21]. The thickness of such sections ranges from 0.3 to 50 µm depending on the procedures applied and is likely to show artefacts such as deformation or the chipping of structures owing to the characteristics of insect cuticle. Furthermore, this method is time consuming and reconstructing structures in three dimensions is demanding.

### 4.2. Computed tomography

Microcomputed tomography (µCT) is a highly efficient technique for studying morphological features. Hörnschemeyer *et al.* [31] established this technique in insect morphology in 2002. µCT combines user friendliness with powerful computational applications to study small- to medium-sized insects. Meanwhile, there are numerous comprehensive studies investigating each tagma of insects: insect head [32,33], insect thorax [27,34], insect abdomen [35,36] and even smaller body parts [5,37]. The advantages of μCT are that it is non-invasive, comparatively rapid, almost free of artefacts, and allow for a three-dimensional reconstruction at convenient nanotomographical resolutions less than 0.1 µm [21] depending on the set-up used for data acquisition. Two studies using μCT resolved the three-dimensional structural organization of the embiopteran spinning apparatus [4,5]. However, as mentioned by Büsse *et al*. [5], the resolution is not adequate to resolve the ultrastructural characters of the syncytial gland cell.

### 4.3. Electron microscopy

TEM is the only appropriate method which allows studying the ultrastructure at cellular level [38]. However, this method shows the same disadvantages as described earlier and in ‘Histology’. Serial section transmission electron microscopy (ssTEM) is challenging, highly time consuming [19] and susceptible to artefacts such as shrinking, distortion and section loss [22].

Fundamental morphological studies of the spinning apparatus of Embioptera were carried out employing TEM [15,16]. These studies supported the multinucleated character of the spinning apparatus cells first mentioned by Mukerji [13]. Furthermore, Alberti & Storch [15] reconstructed the so-called canal cage [14] in detail. However, many questions concerning the morphological organization on cell level had been left unanswered.

SEM is the appropriate method for studying the surface of a sample at high resolution. The visualization of anatomical characters is generally possible but highly limited owing to the need of preparation. Dubitzky & Melzer [17] demonstrate the ejection of silk at the silk ejector of an embiopteran specimen fixated during the spinning process using SEM.

SBFSEM is one of the latest methods introduced to insect morphology [39,40]. It combines an SEM surface scanning with automated ultramicrotome sectioning [19]. Automated data acquisition, minimal artefacts and high resolutions up to cell level, easily suitable for three-dimensional reconstructions are the advantages of this method [26]. However, owing to the currently available hardware configuration, the sample size is somewhat limited as being of 1 × 1 × 1 mm. An insect morphologist would wish for an extended sample size being able to cope with the preparation and the even more demanding sectioning process of insect tissue (e.g. soft tissue most often connected to the extremely hard cuticle). Another disadvantage of SFBSEM is its invasiveness.

In this study, the cell organelles of the silk gland of *Embia* sp. (figures 1–3) can be identified as well as their overall structure, but ultrastructural details of organelles are unidentifiable owing to signal-to-noise ratio and resolution limits, as mentioned in the study by Lipke *et al*. [22] on sperm structure. Initially, these limitations can be attributed to the hardware, but we are continually working on improving the fixation and staining protocol to achieve a better image microcontrast. The step-by-step protocol (electronic supplementary material, S1) represents our latest attempt and will improve signal-to-noise ratio and delineation of structures.

Any and every method has its strengths and limitations. We therefore, suggest a sensible adaptation for every morphological investigation in using a deliberate combination of techniques for the best-possible result. This is not applicable for Embioptera or insects only, but all kinds of organisms, which can be analysed using SBFSEM.

## Supplementary Material

Supplement 1 - SBFSEM step-by-step protocol

## Data accessibility

Used software: Amira^®^ 5.4.3. (FEI SAS, France, www.vsg3d.com) and Adobe Photoshop^®^ CS3 (Adobe System Inc., http://www.adobe.com).

The data supporting this article (electronic supplementary material, S1: step-by-step protocol) have been uploaded as part of the electronic supplementary material. Furthermore, different raw data files (video, tiff-stack, Amira file) are available on dryad (http://www.datadryad.org) under http://dx.doi.org/10.5061/dryad.vk3fg [41].
