# Supplementary material for: Three-dimensional reconstruction on cell level: case study elucidates the ultrastructure of the spinning apparatus of Embia sp. (Insecta: Embioptera)
Source: R Soc Open Sci. 2016 Oct 12;3(10):160563. doi: 10.1098/rsos.160563 (PMC5098999; doi:10.1098/rsos.160563)
Supplement: Supplement 1 - SBFSEM step-by-step protocol [file rsos160563supp1.pdf]

Supplement 1

SBFSEM step-by-step protocol

by Sebastian Büsse, Thomas Hörnschemeyer & Christian Fischer

All steps described in this protocol are realised using a rotary disc/plate and a fume cupboard. Always follow the recommended safety measures of the respective chemical. The precise concentrations and compositions of each chemical used can be found within the 'Material & Methods' section.

Used Chemicals with hazard statements

Glutaraldehyde

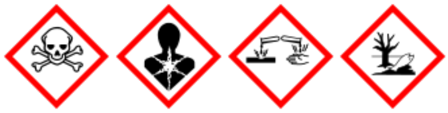

– H302+H332-H314-H317-H334-H335-H410

Sodium cacodylate (rinsing buffer)

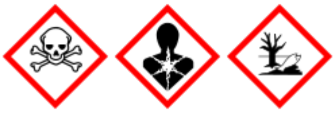

– H301+H331-H351-H410

Osmium tetroxide (OsO_4)

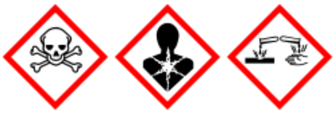

– H300-H310-H314-H330-H334

Potassium hexacyanoferrate – H412

Thiocarbohydrazide (TCH)

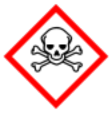

– H300+H330-H311

Uranyl acetate

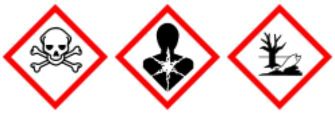

– H330+H300-H373-H411

Lead citrate

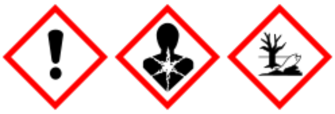

– H302+H332-H360Df-H373-H410

Ethanol (EtOH)

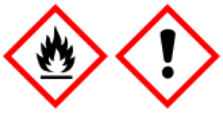

– H225-H319

Acetone

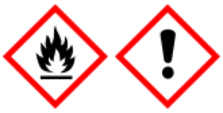

– H225-H319-H336

Durcupan

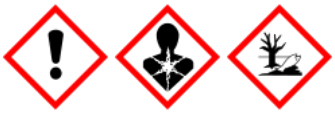

– H302-H315-H317-H319-H360Df-H400-H411

SBFSEM step-by-step protocol

by Sebastian Büsse, Thomas Hörnschemeyer & Christian Fischer

1 — glutaraldehyde
- 4°C
- 90min
- 1x

Sample

Prefixation for 90 min using glutaraldehyde at 4°C

2 — rinsing buffer
- 4°C
- 30sec
- 5x

Sample

Rinsing the sample for 5 times using a cacodylate buffer (rinsing buffer)

3 — OsO_4
- 4°C
- 60min
- 1x

Sample

Staining with osmium tetroxide & potassium hexacyanoferrate for 60 min at 4°C

4 — ddH_2O
- 20°C
- 30sec
- 5x

Sample

Rinsing the sample for 5 times using double-distilled water

5 — TCH
- 20°C
- 20min
- 1x

Sample

Sample fixation with thiocarbohydrazide for 20 min at 20°C

6 — ddH_2O
- 20°C
- 30sec
- 5x

Sample

Rinsing the sample for 5 times using double-distilled water

7 — OsO_4
- 20°C
- ~12h
- 1x

Sample

Staining with osmium tetroxide over night at 20°C (keep dark)

8 — ddH_2O
- 20°C
- 30sec
- 5x

Sample

Rinsing the sample for 5 times using double-distilled water

9 — *new jar
- -
- -
- 1x

Sample

Change the sample jar to a new one

10 — uranyl acetate
- 4°C
- ~12h
- 1x

Sample

Staining with uranyl acetate over night at 4°C

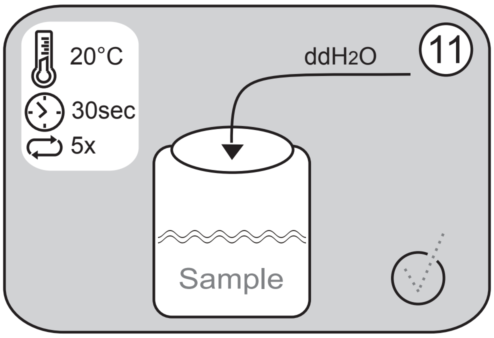

Rinsing the sample for 5 times using double-distilled water

Staining with lead citrate for 30 min at 60°C

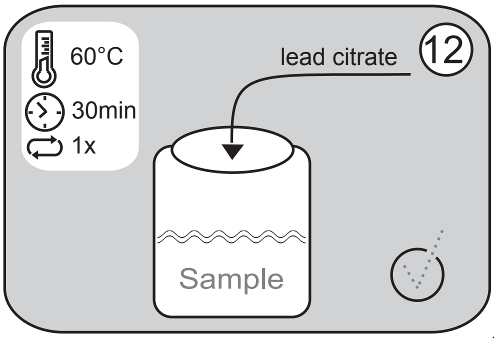

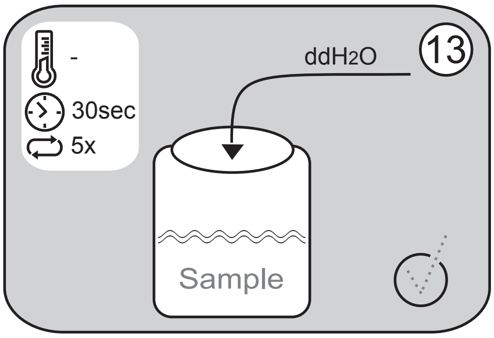

Rinsing the sample for 5 times using double-distilled water

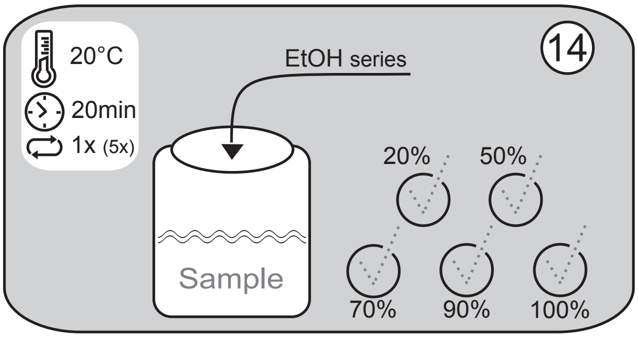

Series of graded ethanol concentrations from 20% to 50% to 70% to 90% to 99,99% for 20 min each

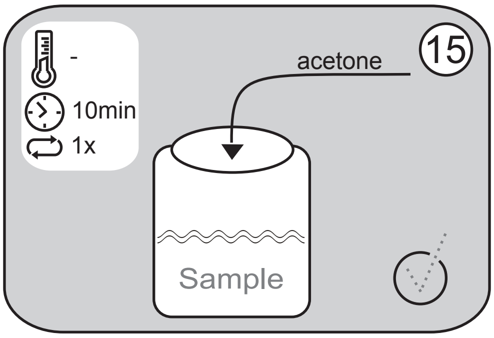

Rinsing for 10 min in acetone

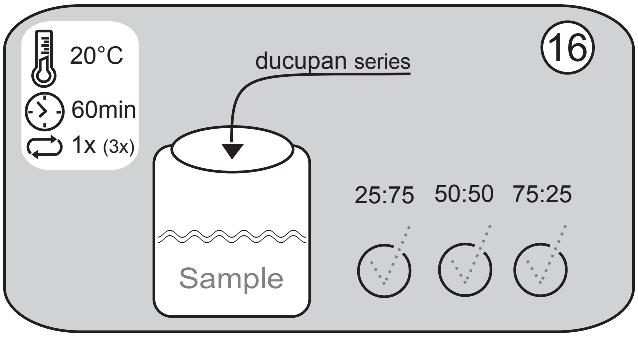

Series of graded durcupan concentrations from 25:75 to 50:50 to 75:50 for 60 min each at 20°C

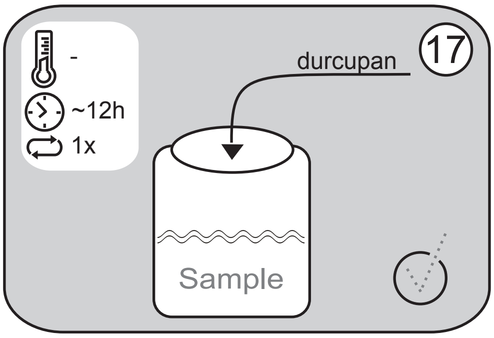

Pure durcupan over night

Fresh pure durcupan
for 10 min

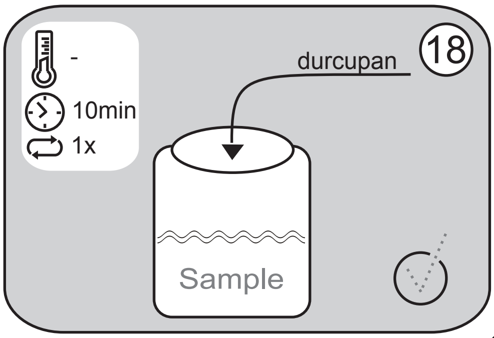

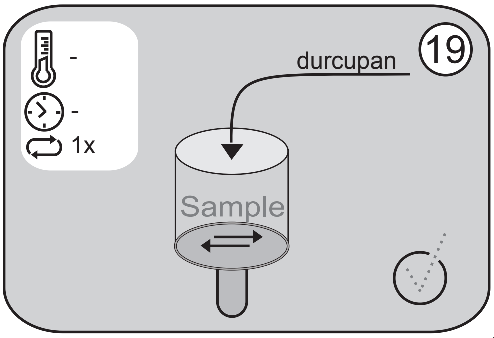

Mounting and alligning of
the sample

Polymerisation of the
embedded sample for at least
48 h at 60°C

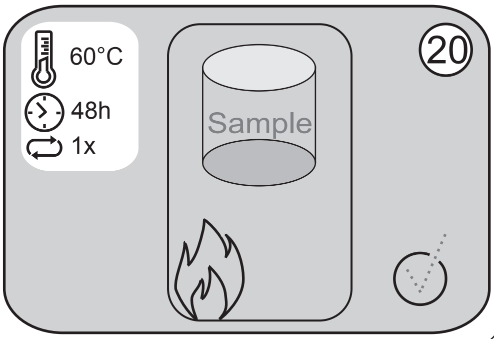

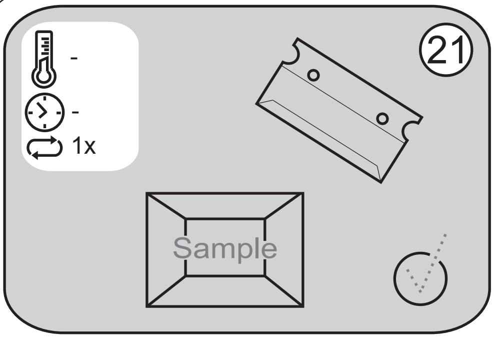

Trimming of the embedde
sample

Sputter coating of the
embedded specimen

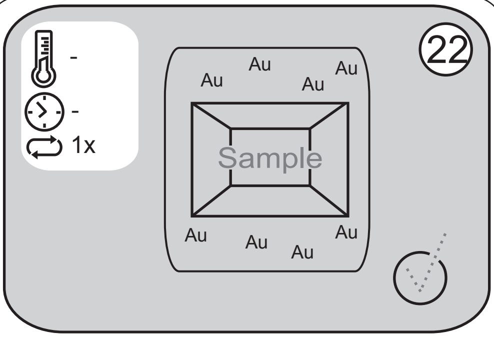

... ready for SBFSEM!
